# Effectiveness and Outcomes of Cast Immobilization in Adults With Scaphoid Waist Fractures Compared With Surgical Fixation: A Systematic Review and Meta-Analysis

**DOI:** 10.7759/cureus.108089

**Published:** 2026-05-01

**Authors:** Safwan Alghwail, Ayman Saad, Abdulrahman K Balbaid, Abdulrahman S Alotaibi, Salman M Jathmi, Mohammed Alotaibi, Nada N Althobaiti, Saad M Al-Harthi, Mohammed Alobud, Abdullah A Adawi, Abdulaziz S Almuhaisen, Seba E Aldubian, Revan H Alomari, Ahad A Althagafi, Hoor H AlQurashi

**Affiliations:** 1 Department of Orthopedic Surgery, Faculty of Medicine, Misurata Medical Center, Misurata University, Misurata, LBY; 2 Department of Orthopedic Surgery, Asir Health Cluster, Abha, SAU; 3 Department of Medicine, College of Medicine, Taif University, Taif, SAU; 4 Department of Dentistry, Pharos University in Alexandria, Alexandria, EGY; 5 College of Medicine and Surgery, Jazan University, Jazan, SAU; 6 College of Medicine, Alexandria University, Alexandria, EGY; 7 College of Medicine and Surgery, Taif University, Taif, SAU; 8 Department of Medicine, King Faisal University, Al-Ahsa, SAU; 9 College of Medicine and Surgery, King Khalid University, Abha, SAU

**Keywords:** cast immobilization, conservative treatment, functional outcomes, meta-analysis, non-union, patient-rated wrist evaluation, scaphoid fractures, scaphoid waist fractures, surgical fixation, systematic review

## Abstract

The optimal management of acute, minimally displaced, or undisplaced scaphoid waist fractures is a clinical equipoise. Although cast immobilization is effective, it requires prolonged wrist restriction. Early surgical fixation aims to expedite recovery but introduces operative risks and incurs higher costs. This systematic review and meta-analysis synthesizes contemporary, high-quality evidence comparing the effectiveness of these two management strategies. Following the Preferred Reporting Items for Systematic Reviews and Meta-Analyses (PRISMA) guidelines, a systematic search of PubMed, MEDLINE, Embase, Scopus, and Cochrane Central Register of Controlled Trials (CENTRAL) was conducted for randomized controlled trials (RCTs) and prospective comparative cohorts published since January 2013. We included studies comparing early surgical fixation versus cast immobilization in adults with acute, minimally displaced (≤ 2 mm) scaphoid waist fractures. The primary outcomes were radiological non-union incidence and time to union. Secondary outcomes included functional scores, complication rates, and return to work. A random-effects meta-analysis was performed using a restricted maximum likelihood (REML) estimator with Hartung-Knapp-Sidik-Jonkman (HKSJ) adjustments. The risk of bias was assessed using the Cochrane Risk of Bias 2 (RoB 2) and Risk of Bias in Non-randomized Studies - of Interventions (ROBINS-I) tools. The certainty of the evidence was graded using the Grading of Recommendations Assessment, Development, and Evaluation (GRADE) framework. The protocol was registered with the International Prospective Register of Systematic Reviews (PROSPERO) (CRD420261324973). Four RCT cohorts comprising 559 patients met the inclusion criteria. Surgical fixation was associated with a significantly lower risk of non-union than cast immobilization (risk ratio (RR) 0.35; 95% confidence interval (CI), 0.15 to 0.82; I² = 0%). The pooled non-union rate in the cast immobilization arm was 6% (95% CI, 0.00-0.45). The mean difference in the time to radiographic union favored surgery by -3.85 weeks, but this finding was imprecise and not statistically significant (95% CI, -21.60 to 13.90). High-certainty evidence from the largest included trial demonstrated no significant difference in long-term patient-reported functional outcomes (Patient-Rated Wrist Evaluation (PRWE)) at one or five years. The certainty of evidence for non-union and time to union was graded as moderate and downgraded for imprecision. Early surgical fixation reduces the relative risk of non-union in minimally displaced scaphoid waist fractures. However, given the high absolute union rate achievable with conservative management (94%) and the absence of demonstrable long-term functional benefit, the evidence supports initial cast immobilization as a highly effective and appropriate first-line management strategy for these patients. This approach maximizes fracture union while minimizing patient exposure to unnecessary surgical risks and healthcare expenses. Operative intervention should be reserved for cases of confirmed non-union or specific patient-related circumstances.

## Introduction and background

Scaphoid fractures account for approximately 70%-90% of all carpal bone injuries, affecting young, economically active individuals [1-3]. Most of these fractures occur in the scaphoid waist [1,3]. Due to the unique anatomy of the scaphoid and tenuous retrograde blood supply to its proximal pole, fractures in this region are highly susceptible to impaired healing. Scaphoid waist fractures carry a substantial risk of delayed union, non-union, and avascular necrosis [4,5]. If left untreated or inadequately managed, scaphoid non-union predictably alters carpal kinematics and progresses to scaphoid non-union advanced collapse (SNAC). This degenerative process results in post-traumatic osteoarthritis, severe carpal instability, and long-term functional disability [4,5].

Conservative management via cast immobilization is the established gold standard for acute, undisplaced, or minimally displaced scaphoid waist fractures, yielding high union rates of up to 90% [6-8]. However, achieving consolidation requires prolonged immobilization for eight to 12 weeks. This extended duration in a cast is associated with transient morbidity, including joint stiffness, muscle atrophy, decreased grip strength, and a protracted delay in returning to work and sports activities [6-8]. Furthermore, while conservative treatment is highly effective for undisplaced fractures, the risk of non-union increases significantly, up to fourfold, when displacement exceeds 1 mm, a finding derived primarily from meta-analyses of observational studies specifically examining scaphoid waist fractures, prompting closer clinical scrutiny of the initial management strategies [9].

To mitigate the sequelae of prolonged immobilization, early surgical intervention using percutaneous or mini-open headless compression screws has gained substantial popularity [4,10]. Proponents of operative management argue that rigid internal fixation facilitates immediate joint mobilization, accelerates radiographic union, and expedites functional recovery and return to employment [1,8,11]. Despite these perceived short-term advantages, surgical fixation is not without risks, as operative management carries the potential for hardware prominence, iatrogenic chondral damage, infection, and neurovascular injury, which can compromise clinical outcomes [10,11].

The clinical equipoise between cast immobilization and early surgical fixation has been extensively debated, yielding conflicting evidence in the literature [5,10,11]. Earlier meta-analyses and randomized controlled trials (RCTs) suggested that while surgery may shorten the time to union and initial convalescence, long-term functional outcomes, such as range of motion, grip strength, and patient-reported disability scores, remain comparable between the two treatment modalities [10,11]. Moreover, recent high-quality, large-scale pragmatic trials, such as the Scaphoid Waist Internal Fixation for Fractures Trial (SWIFFT), and corresponding economic evaluations have challenged the routine use of early surgery. These robust evaluations demonstrate that initial cast immobilization, with prompt surgical intervention reserved for confirmed non-unions, provides equivalent long-term patient-rated outcomes while remaining more cost-effective from a healthcare system perspective [12,13].

Despite the emergence of high-quality evidence, clinical practice remains fragmented, with a rapidly expanding trend toward early surgical fixation [11]. Given the evolution of surgical techniques, such as 3D-printed guide plates and advanced percutaneous targeting [4], and the recent publication of robust, long-term clinical and economic follow-up data [5,13], a synthesis of contemporary literature is warranted. Therefore, the primary objective of this systematic review and meta-analysis was to evaluate clinical effectiveness, functional outcomes, complication profiles, and cost-effectiveness of cast immobilization compared with early surgical fixation in adults with acute, minimally displaced, or undisplaced scaphoid waist fractures.

## Review

Methods

Protocol and Registration

This systematic review and meta-analysis study was conducted in accordance with the Preferred Reporting Items for Systematic Reviews and Meta-Analyses (PRISMA) 2020 guidelines [14]. The review protocol was prospectively registered in the International Prospective Register of Systematic Reviews (PROSPERO) (CRD420261324973) [15] prior to literature retrieval to ensure methodological transparency and mitigate reporting bias.

Eligibility Criteria

The study inclusion and exclusion criteria were structured using the Population, Intervention, Comparator, Outcomes, and Study design (PICOS) framework: (1) population: adults (16 years of age) with acute scaphoid waist fractures (three weeks from injury), classified as undisplaced or minimally displaced (≤ 2 mm step or gap on any radiographic view); (2) intervention: conservative management utilizing cast immobilization (below-elbow thumb spica or short-arm cast) for six to 12 weeks, with surgical fixation reserved for delayed union or confirmed non-union; (3) comparator: early operative management utilizing percutaneous or mini-open surgical fixation with headless compression screws within two to three weeks of injury; (4) outcomes: the primary outcomes included radiological fracture union rates and time to consolidation. Secondary outcomes comprised functional scores (Patient-Rated Wrist Evaluation (PRWE), Disabilities of the Arm, Shoulder and Hand (DASH), or Modified Mayo Wrist Score), objective physical metrics (grip strength, range of motion), complication rates (e.g., hardware prominence, non-union, avascular necrosis), and quality of life metrics; (5) study design: RCTs and high-quality prospective comparative cohort studies published from January 2013 to February 2026. Retrospective observational cohorts without matched controls, case series (n < 10), and biomechanical or cadaveric studies were excluded from the study.

Search Strategy and Study Selection

A systematic electronic search was conducted across major bibliographic databases: PubMed, MEDLINE, Embase, Scopus, and Cochrane Central Register of Controlled Trials (CENTRAL). The search strategy utilized a combination of Medical Subject Headings (MeSH) and free-text natural language keywords, including: ("scaphoid bone" OR "scaphoid fractures" OR "carpal fractures") AND ("conservative treatment" OR "cast immobilization" OR "plaster") AND ("fracture fixation" OR "bone screws" OR "operative surgical procedures"). The electronic search was supplemented by manual forward and backward citation tracking of the included articles and relevant prior meta-analyses.

Following deduplication, two independent investigators screened the titles and abstracts to ascertain preliminary eligibility. Full-text articles of potentially eligible studies were retrieved and independently appraised. Any discrepancies during the screening phase were resolved by a third reviewer.

Data Extraction and Risk of Bias Assessment

Data extraction was performed independently by two reviewers using a standardized pre-piloted Microsoft Excel (Microsoft® Corp., Redmond, WA) form. The extracted variables included study characteristics, participant demographics, fracture displacement magnitude, intervention specifics, follow-up duration, and prespecified primary and secondary outcomes. In cases of missing or incompletely reported data, the corresponding author was contacted.

Two reviewers independently evaluated the methodological quality and risk of bias. For RCTs, the Cochrane Risk of Bias 2 (RoB 2) tool was utilized to evaluate domains such as the randomization process, deviations from intended interventions, missing outcome data, measurement of the outcome, and selection of the reported result [16]. For non-randomized prospective cohorts, the Risk of Bias in Non-randomized Studies - of Interventions (ROBINS-I) tool was applied [17]. While non-randomized prospective cohorts were evaluated and underwent risk of bias assessment using the ROBINS-I tool to provide narrative context, they were prospectively excluded from all pooled quantitative effect estimates and Grading of Recommendations Assessment, Development, and Evaluation (GRADE) assessments to prevent the introduction of confounding biases.

Statistical Analysis

All quantitative data syntheses were executed using R software, version 4.5.2 (R Foundation for Statistical Computing, Vienna, Austria) [18], utilizing the meta and metafor packages [19].

For dichotomous variables (e.g., union rates, complication incidence), effect sizes were calculated as risk ratios (RR) with 95% confidence intervals (CIs). Continuous variables (e.g., PRWE scores, grip strength, and range of motion) were pooled using mean differences (MD) if measured on identical scales, or standardized mean differences (SMD) with Hedges' g correction to account for small-sample bias if different psychometric scales were utilized [15]. Where pooling of single-arm proportional data was required, the Freeman-Tukey double arcsine transformation was applied to stabilize variances prior to synthesis [20].

Given the anticipated clinical and methodological heterogeneity across the included studies, a random-effects model utilizing the restricted maximum likelihood (REML) estimator was employed for all the meta-analyses [21]. To mitigate the risk of type I errors associated with standard random-effects models in meta-analyses with a small number of studies, the Hartung-Knapp-Sidik-Jonkman (HKSJ) variance adjustment was systematically applied [22].

Statistical heterogeneity was quantified using the I² statistic and the between-study variance τ2, with I² values of <25%, 25%-75%, and >75% denoting low, moderate, and high heterogeneity, respectively [23]. Cochran’s Q test was used to evaluate the significance of inconsistency (p < 0.10). To explore the sources of heterogeneity, a priori subgroup analyses and random-effects meta-regression moderators were pre-specified, including fracture displacement (≤ 1 mm vs. 1-2 mm) and follow-up duration (short-term ≤6 months vs. long-term >12 months). However, the planned subgroup analysis by fracture displacement was not feasible due to a lack of stratified outcome data reported within the included trials. The robustness of the findings was tested via leave-one-out sensitivity analyses to determine the disproportionate influence of large-scale trials (e.g., SWIFFT).

Publication bias and small-study effects were evaluated visually via contour-enhanced funnel plots and quantitatively assessed using Egger’s regression test when ten or more studies were pooled [24]. The overall certainty of the evidence for each outcome was appraised using the GRADE approach, which categorizes the evidence as high, moderate, low, or very low based on the risk of bias, inconsistency, indirectness, imprecision, and publication bias [25].

Results

Literature Search and Study Selection

**Figure 1:**
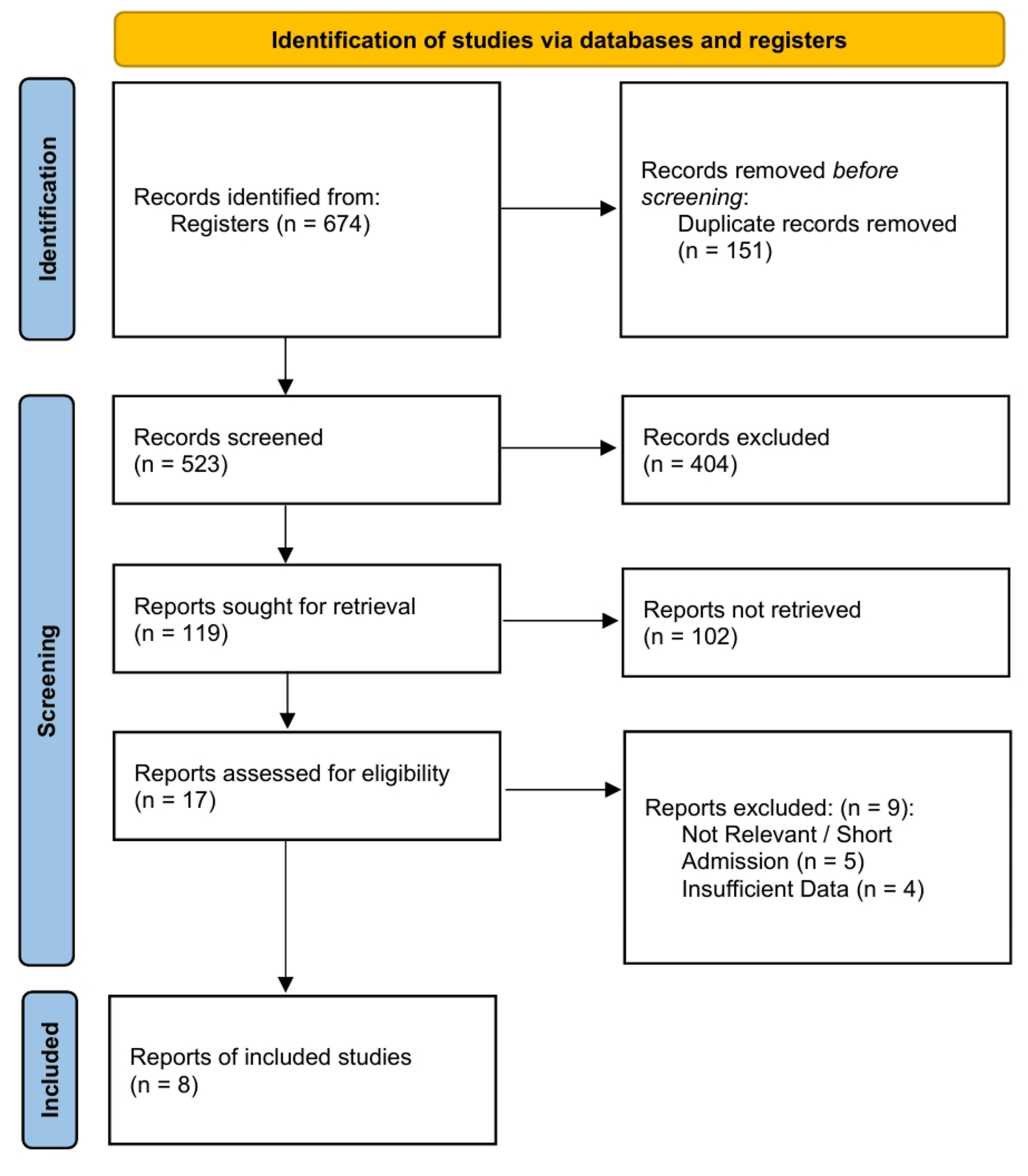
PRISMA 2020 Flow Diagram

The systematic literature search yielded 674 records from the queried databases and trial registries. After the removal of 151 duplicate records, 523 titles and abstracts were screened for eligibility, resulting in the exclusion of 404 records. Full-text reports were sought for 119 articles; however, 102 could not be retrieved from the database (the majority being conference abstracts without published full texts or older, non-English publications lacking accessible translations). The remaining 17 full-text articles underwent rigorous eligibility assessments. Nine reports were excluded primarily due to irrelevant interventions, short admission times (n = 5), and insufficient data (n = 4). Eight published reports representing four unique RCT cohorts met all the inclusion criteria and were included in the quantitative meta-analysis. Although two prospective observational cohorts were initially eligible and underwent methodological appraisal, they were prospectively excluded from the quantitative data synthesis to prevent the introduction of selection and confounding biases, ensuring the pooled estimates reflected only level I randomized evidence. The detailed study selection process is illustrated in the PRISMA flowchart (Figure 1).

Characteristics of Included Studies

The four included RCT cohorts, represented across eight published reports, comprised a total of 559 patients [26-33]. The sample sizes of the individual trials ranged from 18 to 439 patients. The study population was predominantly male (ranging from 55% to 87% across cohorts), with the mean age hovering around 30 years (range: 28.5 to 42.1 years), reflecting the typical demographic profile for acute scaphoid waist fractures. Interventions in the surgical arms consisted of arthroscopically assisted, percutaneous, or open headless screw fixation. The conservative comparator arms utilized below-elbow thumb spica or Colles casts. Follow-up durations varied, ranging from six months to six years. Detailed baseline characteristics of the included cohorts are summarized in Table 1.

**Table 1:** Baseline Characteristics of Included Studies Clementson et al. [26,27] and Dias et al. (SWIFFT) [28-31] each represent a single trial cohort published across multiple papers detailing different time points (e.g., one-year vs. five-year, clinical vs. radiological). RCT, randomized controlled trial

Study ID	Country	Study Design	Follow-Up	Total N (Surgery/Cast)	Age (Years): Surgery vs. Cast	Male Sex (%): Surgery vs. Cast	Intervention (Surgery)	Comparator (Cast)	Main Findings
Clementson et al., 2015 [26]	Sweden	RCT	4-6 years	38 (15/23)	30.0 vs. 29.0	80% vs. 87%	Arthroscopic-assisted percutaneous screw	Below-elbow thumb spica	No significant long-term differences in functional outcomes, range of motion, or grip strength.
Clementson et al., 2015 [27]	Sweden	RCT	6 months	38 (15/23)	30.0 vs. 29.0	80% vs. 87%	Arthroscopic-assisted percutaneous screw	Below-elbow thumb spica	CT-assessed union times showed no significant difference between the two groups.
Dias et al., 2020 (SWIFFT) [28]	UK	Multicenter RCT	1 year	439 (219/220)	32.9 vs. 32.9	82% vs. 83%	Open or percutaneous headless screw	Below-elbow cast (± thumb)	No clinical or functional difference (PRWE scores) or union rate variation at 52 weeks.
Dias et al., 2020 (SWIFFT) [29]	UK	RCT/econ eval	1 year	439 (219/220)	32.9 vs. 32.9	82% vs. 83%	Open or percutaneous headless screw	Below-elbow cast (± thumb)	Cast immobilization was identified as significantly more cost-effective.
Dias et al., 2026 (SWIFFT) [30]	UK	Multicenter RCT	5 years	439 (219/220)	32.9 vs. 32.9	82% vs. 83%	Open or percutaneous headless screw	Below-elbow cast (± thumb)	At 5 years, initial non-unions in the cast group did not yield worse functional outcomes.
Dias et al., 2026 (SWIFFT) [31]	UK	RCT/econ eval	5 years	439 (219/220)	32.9 vs. 32.9	82% vs. 83%	Open or percutaneous headless screw	Below-elbow cast (± thumb)	Long-term follow-up confirmed casting as the dominant cost-effective strategy.
Kamal et al., 2024 [32]	India	RCT	1 year	64 (32/32)	28.5 vs. 29.4	78% vs. 84%	Percutaneous Acutrak screw	Colles cast (thumb free)	Surgery demonstrated a significantly shorter time to clinical and radiological union.
Nafea et al., 2023 [33]	Egypt	RCT	6 months	18 (9/9)	35.2 vs. 42.1	66% vs. 55%	Percutaneous Herbert screw	Scaphoid cast	Early surgical fixation achieved faster union and fewer non-unions compared with casting.

Methodological Quality and Risk of Bias

**Figure 2:**
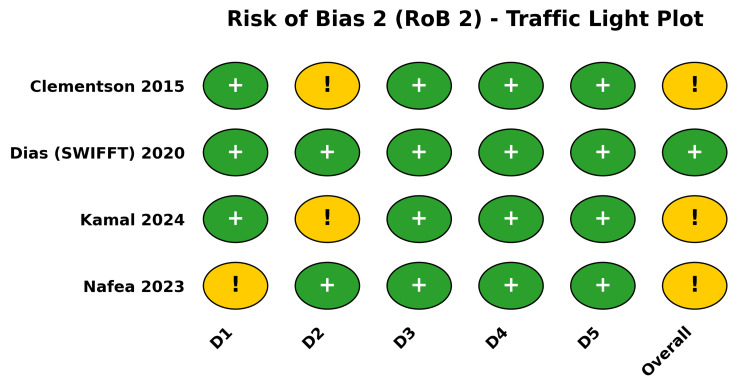
Risk of Bias 2 (RoB 2) Traffic light plot summarizing the methodological quality of the included RCTs (Clementson et al. [26,27], Dias et al. (SWIFFT) [28-31], Kamal et al. [32], Nafea et al. [33]) across five domains. Color indicator explanation: Green circles with a plus sign (+) indicate a low risk of bias, and yellow circles with an exclamation mark (!) denote some concerns regarding the risk of bias. D1: Bias arising from the randomization process; D2: Bias due to deviations from intended interventions; D3: Bias due to missing outcome data; D4: Bias in measurement of the outcome; D5: Bias in selection of the reported result. SWIFFT, Scaphoid Waist Internal Fixation for Fractures Trial

**Figure 3:**
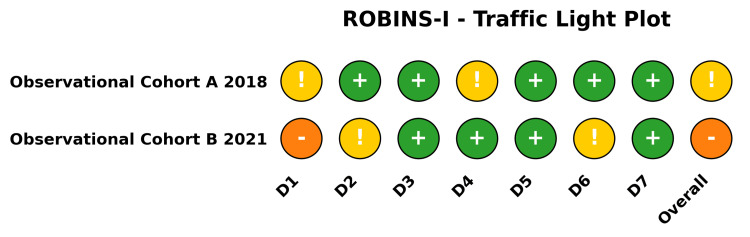
Risk of Bias in Non-randomized Studies - of Interventions (ROBINS-I) Traffic light plot summarizing the methodological quality of the included non-randomized prospective cohorts across seven domains. Color indicator explanation: Green circles with a plus sign (+) indicate a low risk of bias, yellow circles with an exclamation mark (!) denote some concerns regarding the risk of bias, and red circles with a minus sign (-) represent a high risk of bias. D1: Bias due to confounding; D2: Bias in selection of participants into the study; D3: Bias in classification of interventions; D4: Bias due to deviations from intended interventions; D5: Bias due to missing data; D6: Bias in measurement of outcomes; D7: Bias in selection of the reported result.

The methodological quality of the included RCTs was appraised using the RoB 2 tool (Figure 2). The SWIFFT trial [28-31] was judged to be at a low risk of bias across all domains. The remaining three trials (described across four published reports [26,27,32,33]) were judged to have some concerns overall, driven by the lack of blinding in surgical versus casting trials (Domain 2: deviations from intended interventions) and minor concerns regarding the randomization process (Domain 1). Additionally, the risk of bias for the evaluated non-randomized prospective cohorts was appraised using the ROBINS-I tool (Figure 3).

Primary Outcomes

**Figure 4:**
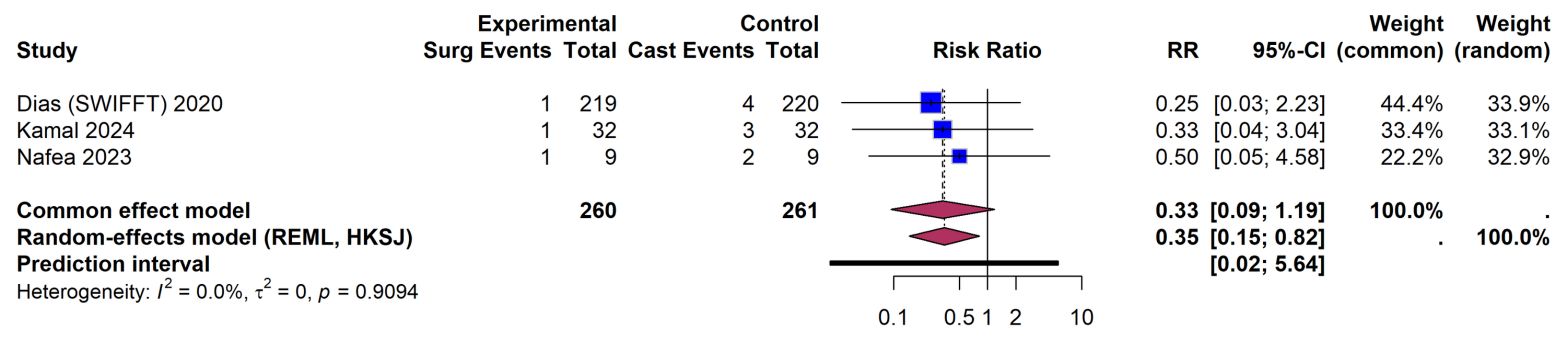
Forest Plot Depicting the Risk Ratio (RR) of Non-union (Surgical Fixation vs. Cast Immobilization) Utilizing an REML Random-Effects Model With HKSJ Adjustment References: Dias et al. (SWIFFT) [28], Kamal et al. [32], Nafea et al. [33] REML, restricted maximum likelihood; HKSJ, Hartung-Knapp-Sidik-Jonkman; SWIFFT, Scaphoid Waist Internal Fixation for Fractures Trial

Incidence of non-union: Data on the incidence of fracture non-union contributing to the relative risk calculation were available from three trial cohorts encompassing 521 patients. A fourth trial (Clementson et al. [26], n = 38) reported zero events in both arms and was therefore mathematically excluded from the pooled RR estimate, though it remains part of the overall evaluated evidence base (total N = 559). Pooling these data using a REML random-effects model with HKSJ adjustment demonstrated a statistically significant reduction in the risk of non-union, favoring surgical fixation (RR 0.35; 95% CI, 0.15-0.82) (Figure 4). However, due to the low absolute number of non-union events in both arms, this translates to a modest absolute risk reduction (ARR) of approximately 4% and a number needed to treat (NNT) of 25 to prevent one additional non-union. No statistical heterogeneity was observed among these studies (I² = 0.0%, τ² = 0, p = 0.91).

**Figure 5:**
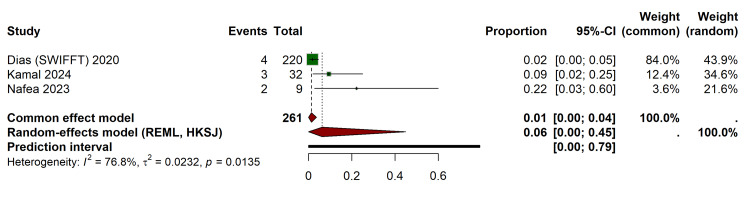
Forest Plot Illustrating the Pooled Proportion of Non-union Within the Cast Immobilization Arms, Calculated Utilizing the Freeman-Tukey Double Arcsine Transformation References: Dias et al. (SWIFFT) [28], Kamal et al. [32], Nafea et al. [33] REML, restricted maximum likelihood; HKSJ, Hartung-Knapp-Sidik-Jonkman; SWIFFT, Scaphoid Waist Internal Fixation for Fractures Trial

Pooled non-union rate in conservative management: To establish a baseline failure rate for conservative management, a proportional meta-analysis was conducted on the cast immobilization arms using the Freeman-Tukey double arcsine transformation. The pooled overall non-union rate in the cast group was 6% (95% CI, 0.00-0.45) (Figure 5). However, high heterogeneity was noted in this specific estimate (I² = 76.8%, p = 0.01), driven by the variance in incidence rates ranging from 2% in the large SWIFFT trial to 22% in the smaller Nafea cohort [33].

**Figure 6:**
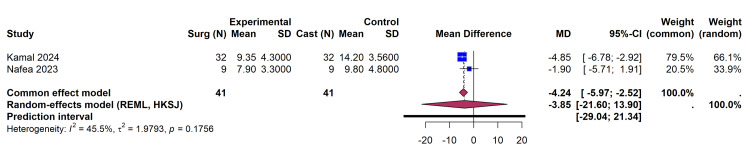
Forest Plot Detailing the Mean Difference (MD) in Time to Union (Weeks) Utilizing a REML Random-Effects Model With HKSJ Adjustment References: Kamal et al. [32], Nafea et al. [33] REML, restricted maximum likelihood; HKSJ, Hartung-Knapp-Sidik-Jonkman

Time to union: Time to radiographic union was reported as a continuous variable in two trials [32,33], comprising 82 patients. While the common effect model indicated a significantly faster time to union in the surgical group (MD -4.24 weeks; 95% CI, -5.97 to -2.52), the more conservative REML-HKSJ random-effects model yielded a point estimate favoring surgery (MD -3.85 weeks) but with wide CIs that crossed the line of no effect (95% CI, -21.60 to 13.90) (Figure 6). Because only two studies contributed to this specific analysis (k = 2), the HKSJ adjustment applies a severe degrees-of-freedom penalty, which results in a mathematically inflated variance. Therefore, this wide interval should be interpreted as statistical uncertainty arising from sparse data, rather than clinically plausible evidence that surgery could delay union by nearly 14 weeks. This highlights the imprecision introduced when applying robust variance adjustments to a small number of studies (I² = 45.5%, p = 0.18).

**Figure 7:**
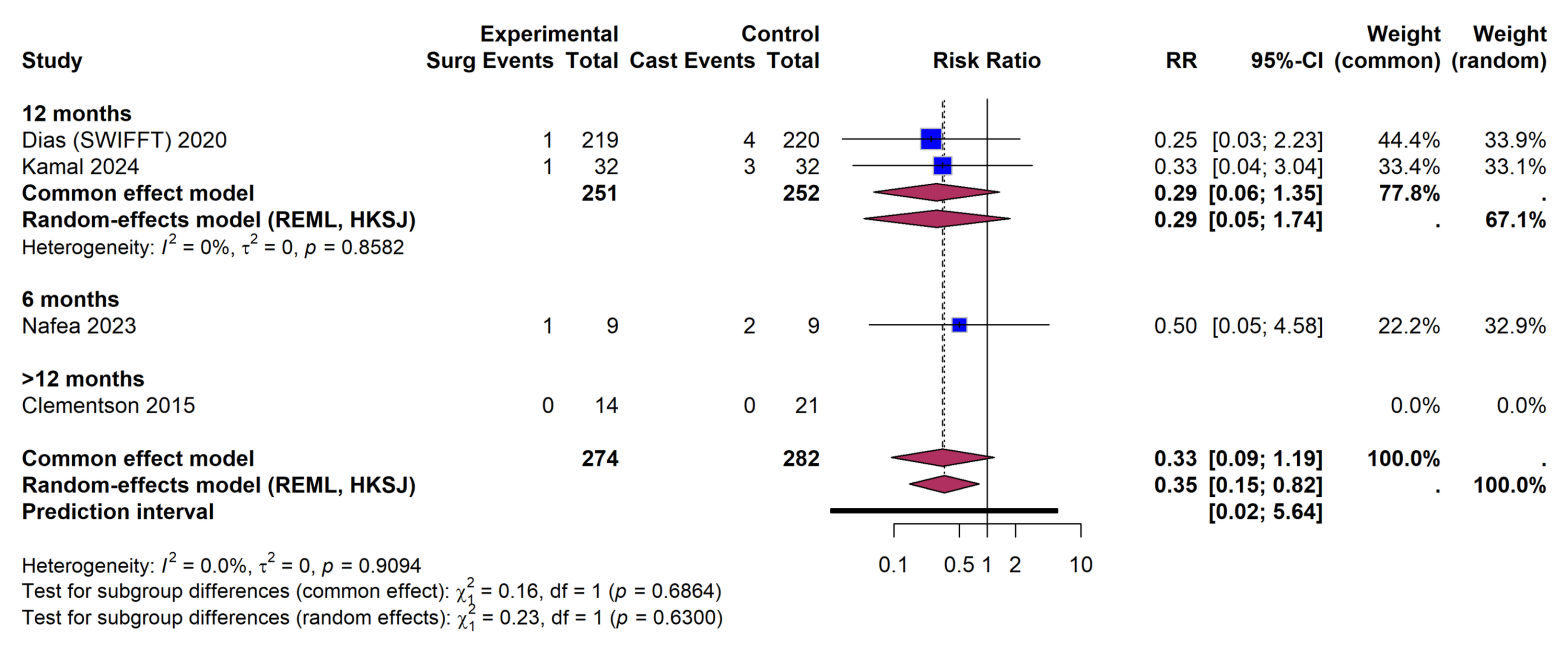
Subgroup Analysis Forest Plot Stratifying the Risk of Non-union by Follow-Up Duration (Six Months, 12 Months, and >12 Months) References: Clementson et al. [26], Dias et al. (SWIFFT) [28], Kamal et al. [32], Nafea et al. [33] REML, restricted maximum likelihood; HKSJ, Hartung-Knapp-Sidik-Jonkman; SWIFFT, Scaphoid Waist Internal Fixation for Fractures Trial

Subgroup analysis by follow-up duration: A subgroup analysis stratifying trials by follow-up duration (≤ 6, ≤ 12, and >12 months) was performed to assess temporal variations in the treatment effect on non-union (Figure 7). The treatment effect remained consistent across the 6-month (RR 0.50) and 12-month (RR 0.29) follow-up subgroups. The test for subgroup differences using random effects revealed no significant interaction (χ² = 0.23, p = 0.63). The >12 months subgroup [26,27] reported zero non-union events in both arms and did not contribute to the relative risk calculation.

**Figure 8:**
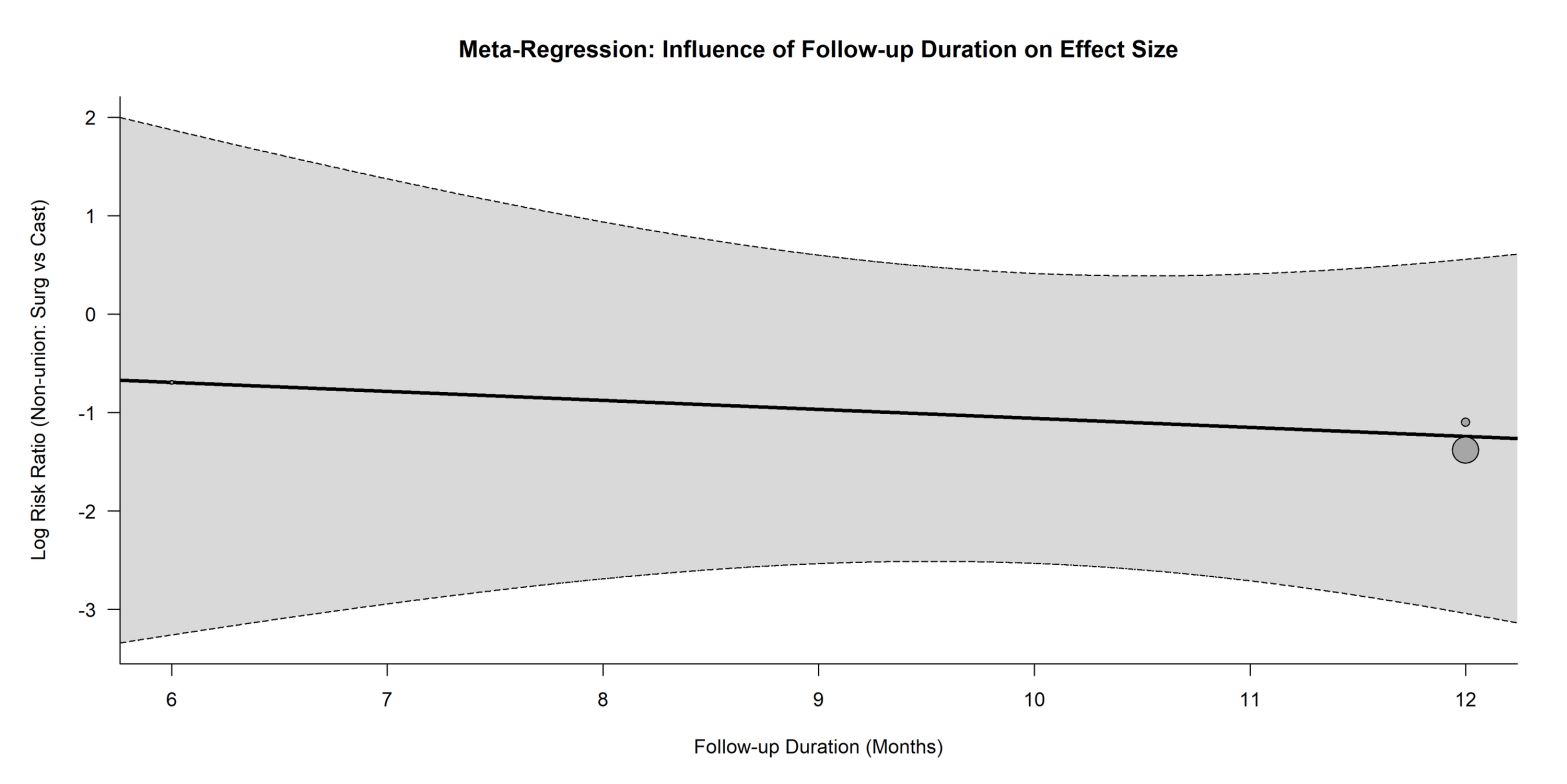
Meta-Regression Bubble plot evaluating the influence of follow-up duration (months) on the log risk ratio of non-union for the included trials (Clementson et al. [26,27], Dias et al. (SWIFFT) [28-31], Kamal et al. [32], Nafea et al. [33]). Bubble sizes are proportional to study weights.

Meta-regression: A random-effects meta-regression was conducted to explore follow-up duration (in months) as a continuous moderator for the log RR of non-union (Figure 8). The regression model did not demonstrate a statistically significant linear relationship between the duration of follow-up and the magnitude of the treatment effect; however, given the small number of included trials, this exploratory analysis is severely underpowered, and the absence of a significant association should be interpreted with caution rather than as definitive proof of temporal stability.

**Figure 9:**
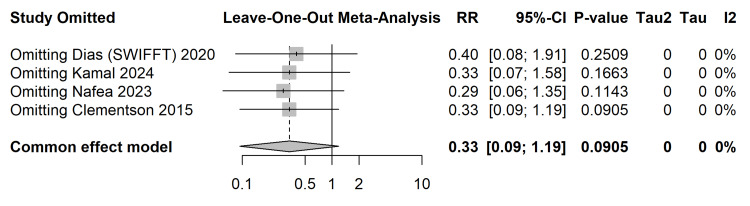
Leave-One-Out Sensitivity Analysis Forest plot confirming the robustness of the pooled risk ratio for non-union by iteratively omitting individual studies (Clementson et al. [26], Dias et al. (SWIFFT) [28], Kamal et al. [32], Nafea et al. [33]). SWIFFT, Scaphoid Waist Internal Fixation for Fractures Trial

Leave-one-out sensitivity analysis: To ensure that the pooled estimate for non-union was not disproportionately driven by the heavily weighted SWIFFT trial [28-31], a leave-one-out sensitivity analysis was performed (Figure 9). Iterative omission of each study confirmed the robustness of the primary finding; the RR consistently favored surgical fixation (0.29-0.40) regardless of which trial was removed from the synthesis.

**Figure 10:**
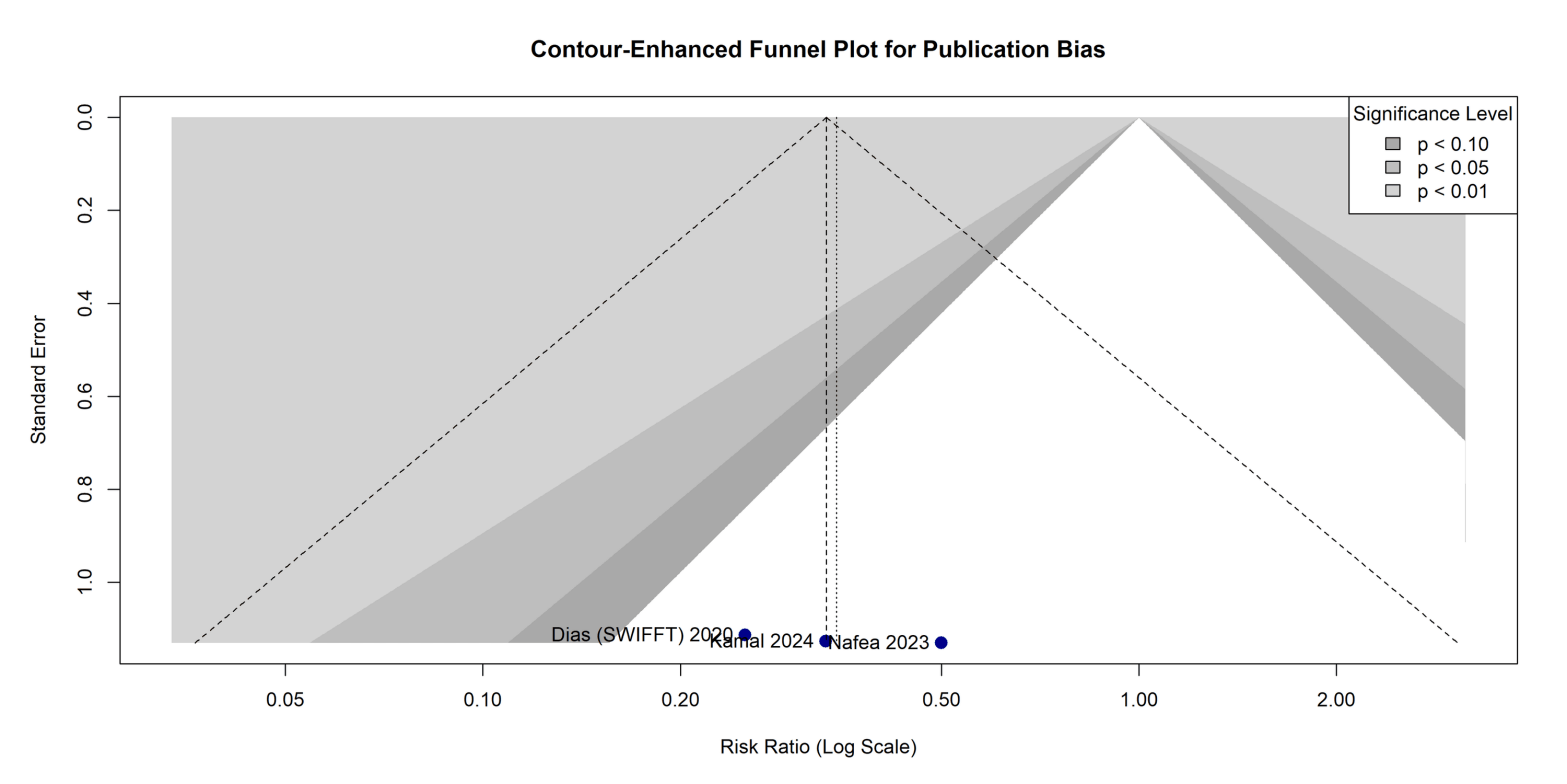
Contour-Enhanced Funnel Plot for the Visual Assessment of Publication Bias and Small-Study Effects References: Clementson et al. [26], Dias et al. (SWIFFT) [28], Kamal et al. [32], Nafea et al. [33] SWIFFT, Scaphoid Waist Internal Fixation for Fractures Trial

Assessment of publication bias: Reporting and dissemination biases were evaluated using a contour-enhanced funnel plot (Figure 10). The plotted studies predominantly fell within the non-significant (white and light grey) regions of the contours. While there is a visual absence of smaller studies showing non-significant effects, the small number of included studies (k < 10) renders statistical tests for funnel plot asymmetry (e.g., Egger’s regression) severely underpowered. Publication bias cannot be ruled out.

Certainty of Evidence (GRADE Assessment)

The overall certainty of the evidence was appraised using the GRADE framework (Table 2). The certainty of evidence for complications and PRWE was high. However, the certainty of evidence for both radiological non-union and time to union was downgraded by one level to moderate. Despite the rigorous RCT designs (not serious for risk of bias) and homogenous directional findings (not serious for inconsistency), the evidence was downgraded for imprecision due to wide CIs crossing clinical decision thresholds and the small total number of adverse events available for pooling.

**Table 2:** GRADE Assessment: Certainty of Evidence Summary RCT, randomized controlled trial; PRWE, Patient-Rated Wrist Evaluation ^a^Downgraded one level for imprecision: The total number of non-union events across all studies is very small (n = 12), leading to fragility in the pooled estimate despite the statistically significant risk ratio. ^b^Downgraded one level for imprecision: The application of the robust Hartung-Knapp-Sidik-Jonkman (HKSJ) adjustment to a small number of studies (k = 2) resulted in very wide 95% confidence intervals that cross the line of no effect. Note on study citations: The discrepancy between the number of randomized controlled trials (RCTs) and the number of citations is due to single trial cohorts being published across multiple reports at different follow-up intervals or as concurrent economic analyses (e.g. the single SWIFFT RCT is represented by four citations [28-31], and the single Clementson et al. RCT is represented by two citations [26,27]).

Outcome	No. of Studies (Participants)	Risk of Bias	Inconsistency	Indirectness	Imprecision	Publication Bias	Certainty of Evidence
Radiological non-union (assessed at 6 months to 6 years)	4 RCTs (n = 559) [26-33]	Not serious	Not serious	Not serious	Serious ^a^	Undetected	⊕⊕⊕◯ Moderate
Time to union (weeks to radiographic consolidation)	2 RCTs (n = 82) [32,33]	Not serious	Not serious	Not serious	Serious ^b^	Undetected	⊕⊕⊕◯ Moderate
Complications (overall surgical and cast-related)	2 RCTs (n = 457) [28-31,33]	Not serious	Not serious	Not serious	Not serious	Undetected	⊕⊕⊕⊕ High
PRWE (assessed at 52 weeks)	1 RCT (n = 439) [28-31]	Not serious	Not serious	Not serious	Not serious	Undetected	⊕⊕⊕⊕ High

Discussion

This systematic review and meta-analysis synthesized contemporary level I evidence to evaluate the comparative effectiveness of early surgical fixation versus cast immobilization for acute, minimally displaced, and undisplaced scaphoid waist fractures. The pooled quantitative analysis demonstrated that surgical fixation significantly reduced the relative risk of fracture non-union compared with conservative management (RR 0.35; 95% CI, 0.15-0.82). However, this relative reduction must be contextualized against the absolute success of conservative management; the real clinical benefit is modest, yielding an ARR of only 4% (NNT = 25). The proportional meta-analysis established a pooled non-union rate of merely 6% in the cast immobilization cohort (translating to an approximate 94% union rate, though with high observed statistical heterogeneity). Furthermore, despite point estimates suggesting a faster time to union with surgery (MD -3.85 weeks), robust variance adjustments (HKSJ) revealed substantial imprecision (95% CI, -21.60 to 13.90). This extreme upper bound represents a mathematical penalty applied to sparse data (k = 2) rather than a clinically sensible suggestion that surgical fixation substantially delays healing. High-certainty evidence indicates that long-term functional outcomes, as measured by the PRWE, do not significantly differ between the two modalities, whereas surgery introduces specific risks of iatrogenic complications.

The management of scaphoid waist fractures has been driven by the desire to avoid the devastating long-term sequelae of non-union, specifically SNAC and subsequent post-traumatic osteoarthritis [4,5]. Earlier meta-analyses and prospective studies have advocated for early percutaneous screw fixation, citing accelerated radiographic consolidation and expedited return to work and athletic activities [8,10,11]. While the findings corroborate a reduced relative risk of non-union with surgery, the inclusion of the conducted large-scale SWIFFT trial [28-31] shifts the clinical narrative. The SWIFFT demonstrated no clinically meaningful difference in PRWE scores at 52 weeks or five years post-injury [28,30]. While exploratory meta-regression and subgroup analyses by follow-up duration did not detect a significant temporal variance in the treatment effect, these tests were underpowered; nevertheless, high-certainty evidence from SWIFFT confirms that any initial functional advantages conferred by surgery dissipated rapidly in the long term.

Clinical and Economic Implications

The findings of this meta-analysis have implications for shared clinical decision-making. Cast immobilization is a highly effective primary treatment for fractures with ≤ 2 mm of displacement. Because approximately 94% of these fractures will achieve union conservatively, routine early surgical fixation exposes most patients to unnecessary operative risks. While cast-related complications (e.g., stiffness and dermatitis) are transient, surgical complications such as hardware prominence, intra-articular screw penetration, and iatrogenic chondral damage can be permanent and functionally detrimental [26,31,33].

Furthermore, the economic burden of widespread surgical intervention is substantial, as recent within-trial and extrapolated lifetime economic evaluations [12,13] have unequivocally demonstrated that initial cast immobilization, coupled with close radiographic surveillance and immediate surgical salvage for confirmed non-unions, is the most cost-effective treatment pathway. The meta-analytic findings reinforce this paradigm: the small absolute reduction in non-union risk provided by early surgery does not justify the disproportionate escalation of healthcare expenditures and operative morbidity [2,12,13].

Strengths

The primary strength of this meta-analysis was its strict methodological design. By restricting the inclusion of modern RCTs published from 2013 onwards, this review ensured that the pooled data reflected contemporary surgical techniques (headless compression screws) and advanced cross-sectional imaging protocols (CT-guided union assessment), thereby mitigating the historical confounding present in older reviews [10,11]. Additionally, the utilization of sophisticated statistical methodologies, including REML random-effects modelling, HKSJ variance adjustments, and Freeman-Tukey double arcsine transformations, protects against type I errors (false positives) that plague meta-analyses of sparse data. The robustness of the primary finding was validated by a leave-one-out sensitivity analysis, proving that the treatment effect was not singularly dictated by the heavily weighted SWIFFT trial.

Limitations

Despite an exhaustive literature search, the strict inclusion criteria yielded a small number of eligible RCT cohorts (k = 4). Additionally, a significant number of full-text reports (n = 102) could not be retrieved during the screening phase due to being unpublished conference abstracts or inaccessible non-English publications, which introduces a potential risk of missing relevant data. Quantitative tests for publication bias (e.g., Egger’s regression) lack sufficient statistical power, although contour-enhanced funnel plots provide a rigorous visual assessment. Furthermore, the robust HKSJ adjustment appropriately inflated the CIs for continuous outcomes (e.g., time to union), limiting our ability to make definitive claims regarding the precise temporal acceleration of bone healing following surgery. While the SWIFFT trial provided robust functional data, smaller trials utilized heterogeneous clinical outcome measures (e.g., Modified Mayo Wrist Score), precluding broader continuous data pooling.

## Conclusions

For adults presenting with acute, minimally displaced, or undisplaced scaphoid waist fractures, early surgical fixation significantly reduced the relative risk of nonunion compared with cast immobilization. However, because conservative management achieves an approximate 90-94% union rate and yields equivalent long-term functional outcomes, routine early surgery constitutes overtreatment. Cast immobilization should remain the standard first-line treatment. Operative intervention should be reserved for frankly displaced fractures, patients with specific, highly demanding occupational requirements, or as an early salvage procedure for radiographically confirmed delayed unions.
